# Supplementary material for: A biological camera that captures and stores images directly into DNA
Source: Nat Commun. 2023 Jul 3;14:3921. doi: 10.1038/s41467-023-38876-w (PMC10318082; doi:10.1038/s41467-023-38876-w)
Supplement: Supplementary file 1 — Supplementary Information [file 41467_2023_38876_MOESM1_ESM.pdf]

Supplementary File

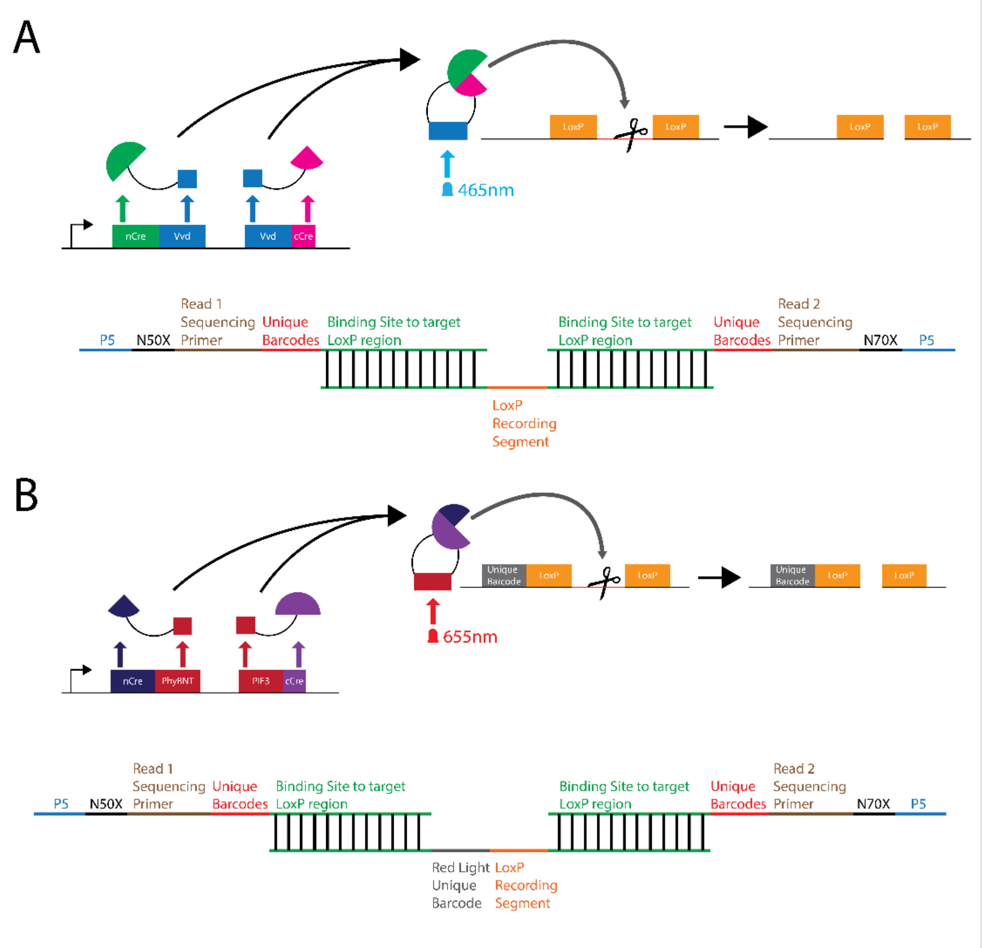

Supplementary S1: Biochemical basis of optogenetic recombination and barcoding of sequencing product. **A**: Blue light Cre-recombinase mode of action and final sequencing product. **B**: Red light Cre-recombinase mode of action and final sequencing product. The unique red light barcode sequence preceding the LoxP recording sequence allows for differentiation between red and blue light sensitive populations.

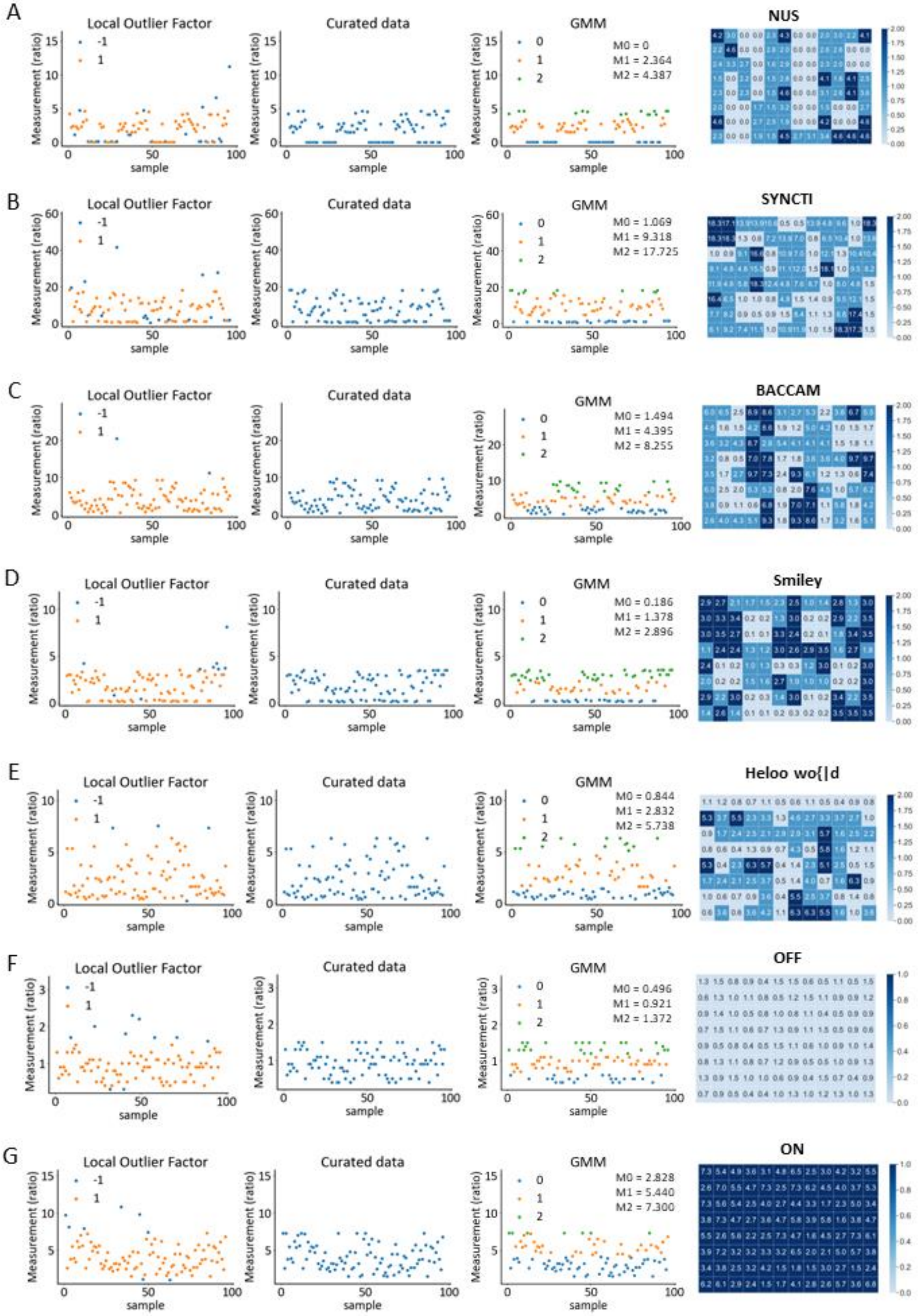
A
Local Outlier Factor
Curated data
GMM
M0 = 0
M1 = 2.364
M2 = 4.387
NUS
B
Local Outlier Factor
Curated data
GMM
M0 = 1.069
M1 = 9.318
M2 = 17.725
SYNCTI
C
Local Outlier Factor
Curated data
GMM
M0 = 1.494
M1 = 4.395
M2 = 8.255
BACCAM
D
Local Outlier Factor
Curated data
GMM
M0 = 0.186
M1 = 1.378
M2 = 2.896
Smiley
E
Local Outlier Factor
Curated data
GMM
M0 = 0.844
M1 = 2.832
M2 = 5.738
Heloo wo{|d
F
Local Outlier Factor
Curated data
GMM
M0 = 0.496
M1 = 0.921
M2 = 1.372
OFF
G
Local Outlier Factor
Curated data
GMM
M0 = 2.828
M1 = 5.440
M2 = 7.300
ON
Measurement (ratio)
sample

Supplementary S2:
Implementing the automated workflow using 3-component Gaussian Mixture Model (GMM) as clustering technique for Image deconvolution. A-E: Results for the five different patterns after outlier detection (first column) and reassignment (second column), the computed means (M0-M2) for the individual clusters after implementing the clustering technique (third column), and the corresponding heatmap plots before cluster grouping (fourth column). F-G: Results for capturing full ‘OFF’ and ‘ON’ data after assessing the satisfaction of the highest mean and lowest mean against the ON-OFF threshold respectively and reassigning label to all 0s (OFF) or 1s (ON), and the respective heatmaps.

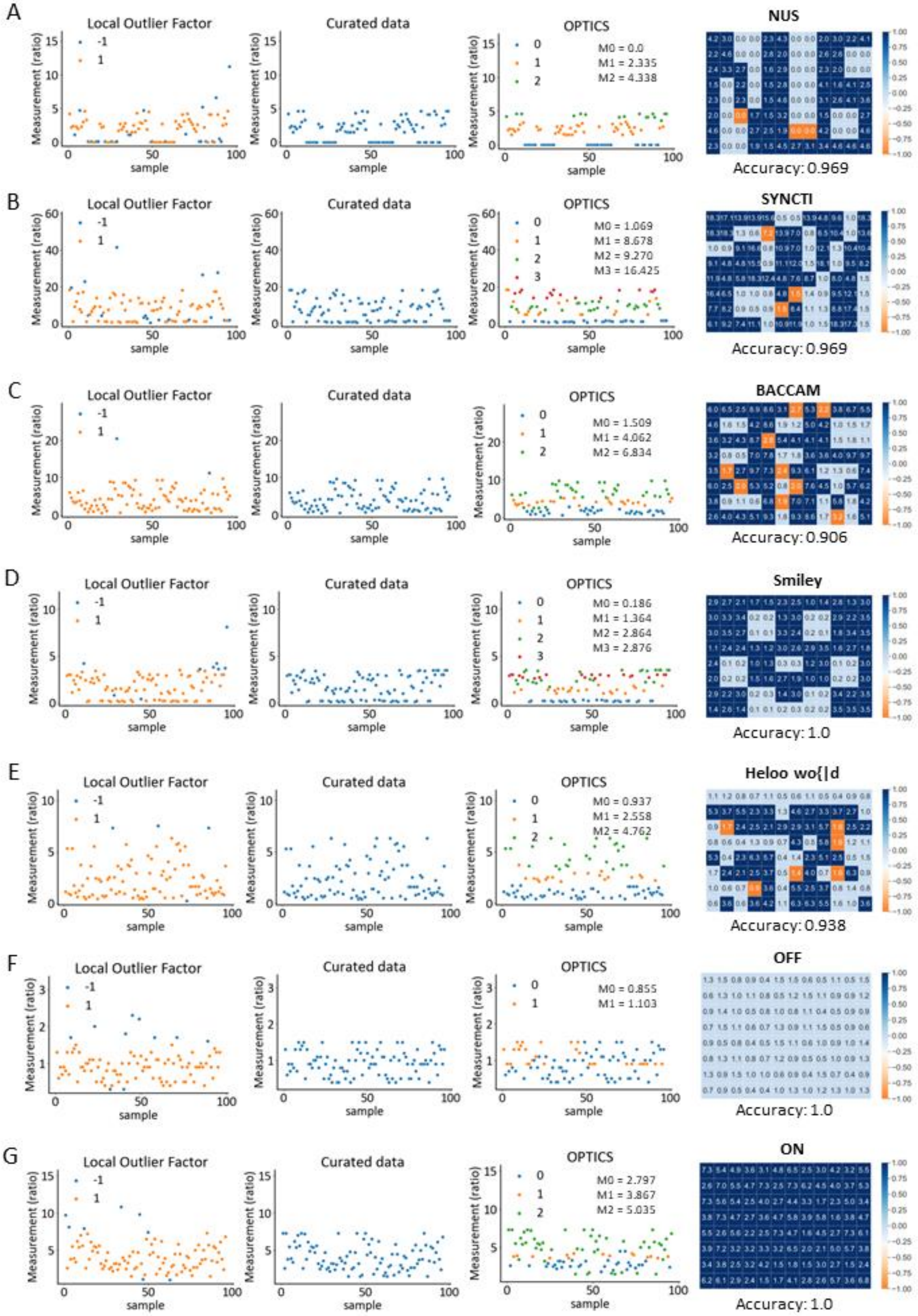

A
Local Outlier Factor
-1
1
Curated data
OPTICS
M0 = 0.0
M1 = 2.335
M2 = 4.338
NUS
Accuracy: 0.969
B
Local Outlier Factor
Curated data
OPTICS
M0 = 1.069
M1 = 8.678
M2 = 9.270
M3 = 16.425
SYNCTI
Accuracy: 0.969
C
Local Outlier Factor
Curated data
OPTICS
M0 = 1.509
M1 = 4.062
M2 = 6.834
BACCAM
Accuracy: 0.906
D
Local Outlier Factor
Curated data
OPTICS
M0 = 0.186
M1 = 1.364
M2 = 2.864
M3 = 2.876
Smiley
Accuracy: 1.0
E
Local Outlier Factor
Curated data
OPTICS
M0 = 0.937
M1 = 2.558
M2 = 4.762
Heloo wo{|d
Accuracy: 0.938
F
Local Outlier Factor
Curated data
OPTICS
M0 = 0.855
M1 = 1.103
OFF
Accuracy: 1.0
G
Local Outlier Factor
Curated data
OPTICS
M0 = 2.797
M1 = 3.867
M2 = 5.035
ON
Accuracy: 1.0
Measurement (ratio)
sample

Supplementary S3:
Implementing the automated workflow using OPTICS as clustering technique for image deconvolution. A-E: Results for the five different patterns after applying Local Outlier Factor to detect outliers (‘1’: inliers and ‘-1’: outliers), curated data obtained after outlier reassignment to the nearest inlier value, computed means (M0-M3) for the individual clusters after implementing clustering technique and the corresponding heatmap plots after cluster grouping. The pixels with value -1 (orange) were used to signify the inaccurate pixels when comparing with the true values. F-G: Results for capturing full ‘OFF’ and ‘ON’ data after assessing the satisfaction of the highest mean and lowest mean against the ON-OFF threshold respectively and label reassignment to all 0s (OFF) or 1s (ON), and the heatmap plots.

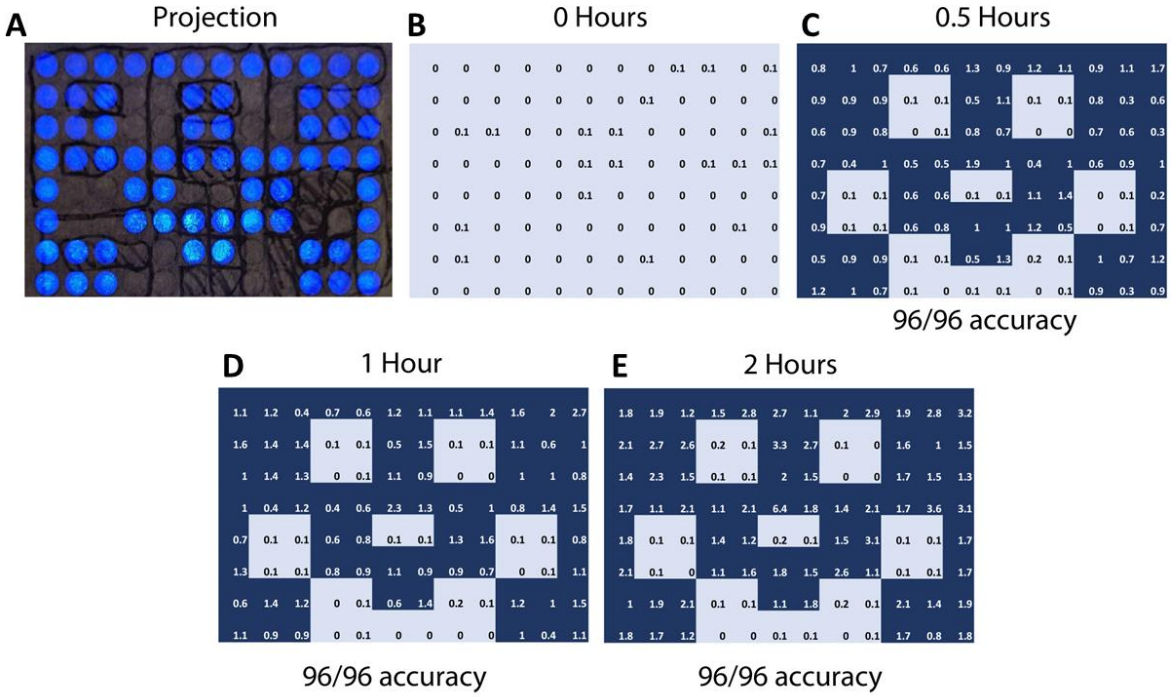

Supplementary S4:
Exposure time experiment demonstrating the kinetics of image encoding. Cells were aliquoted out for colony PCR and barcoding at various intervals to encode images. **A**: Image to be encoded for this experiment. **B-E**: Images obtained after PCR and barcoding at increasing exposure time intervals.

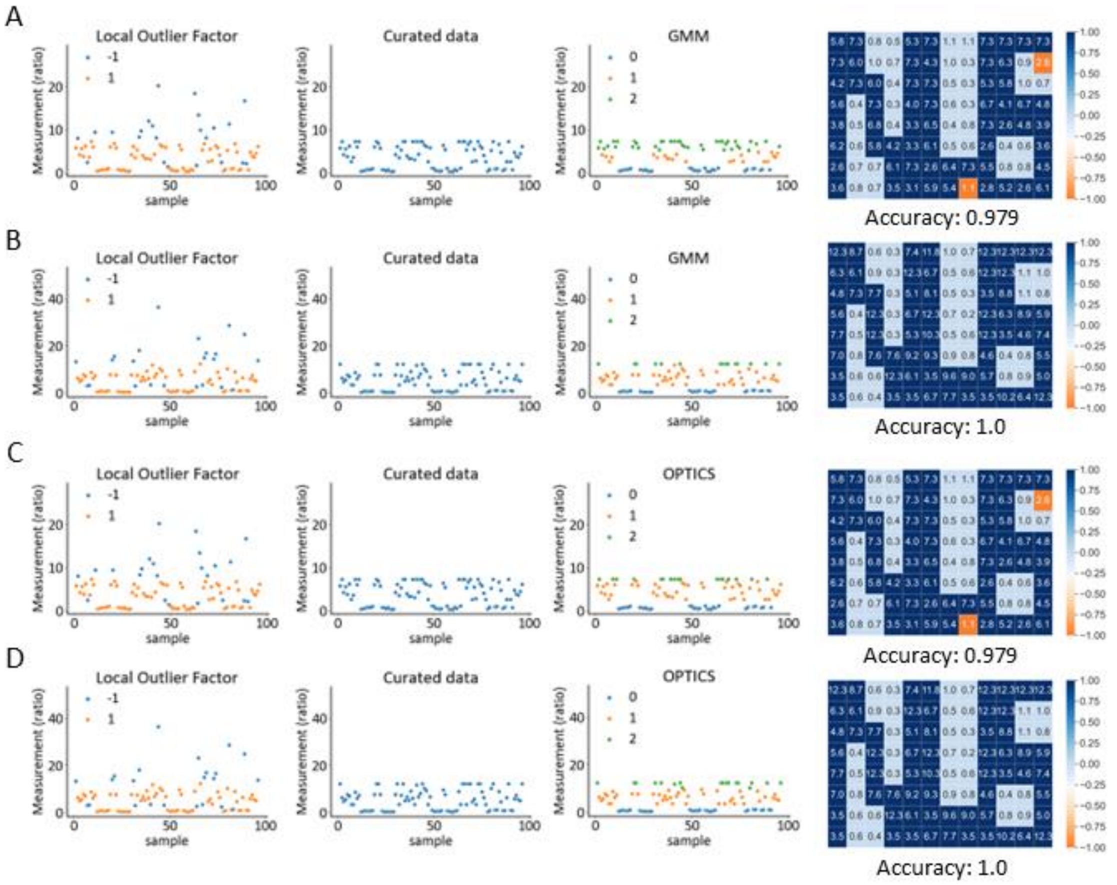

Supplementary S5:
Implementing the automated workflow for the blue-light pattern deconvolution when projecting in an alternating fashion (**A**, **C**) and under simultaneous projection (**B**, **D**). **A-B**: Using 3-component Gaussian Mixture Model (GMM) as the clustering technique for deconvoluting the two blue-light patterns. **C-D**: Using OPTICS as the clustering technique for deconvoluting the two blue-light patterns. The pixels with value -1 (orange) were used to signify the inaccurate pixels when comparing with the true values.

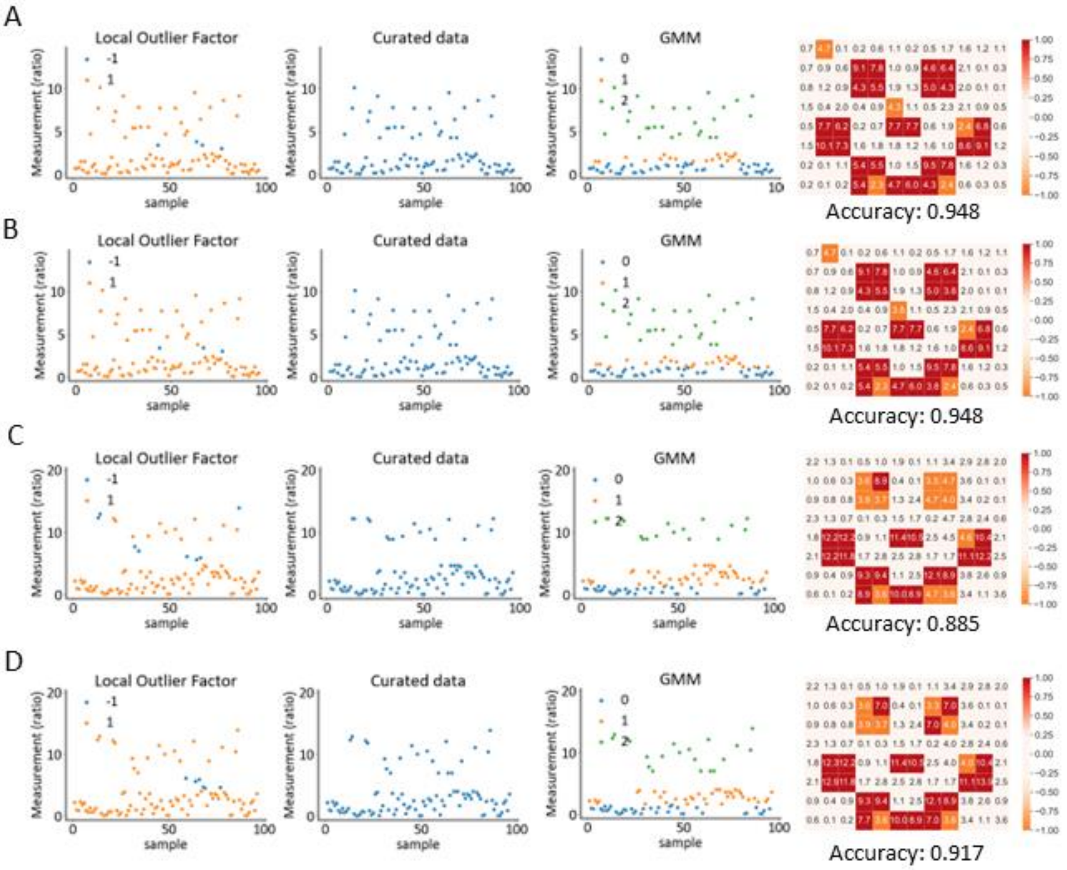

Supplementary S6:
Optimizing the parameter (n_neighbors) of outlier detection algorithm for deconvoluting red-light patterns using 3-component Gaussian Mixture Model (GMM). **A-B**: Performance for the red-light pattern deconvolution when projecting in an alternating fashion after applying (**A**) n_neighbors = 20 or (**B**) n_neighbors = 10 to Local Outlier Factor when detecting outliers. **C-D**: Performance for the red-light pattern deconvolution under simultaneous projection when applying (**C**) n_neighbors = 20 or (**D**) n_neighbors = 10 to Local Outlier Factor. The pixels with value -1 (orange) were used to signify the inaccurate pixels when comparing with the true values. Note: For red-light pattern deconvolution, cluster grouping was executed by grouping the two clusters with lower means into a single ‘OFF’ cluster and the other cluster/s as ‘ON’ cluster.

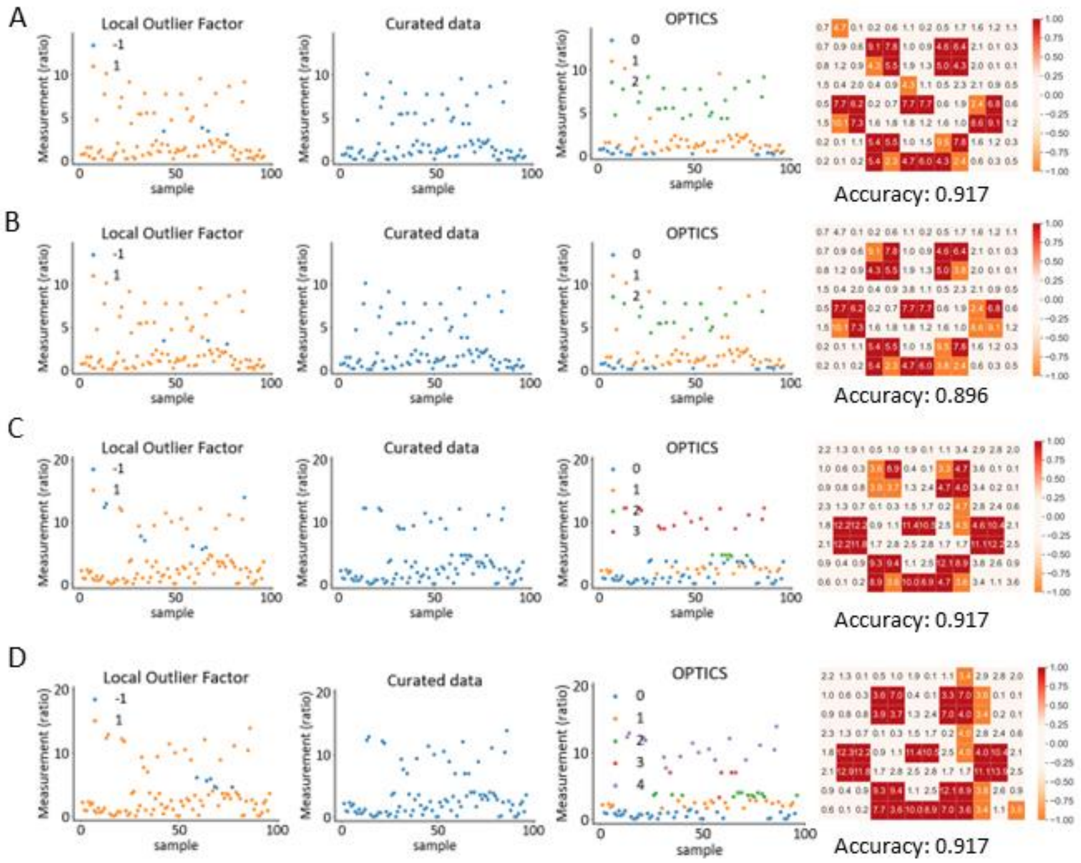

Supplementary S7:
Testing the parameter (n_neighbors) of outlier detection algorithm for deconvoluting red-light patterns using OPTICS. **A-B**: Performance for the red-light pattern deconvolution when projecting in an alternating fashion after applying (**A**) n_neighbors = 20 or (**B**) n_neighbors = 10 to Local Outlier Factor when detecting outliers. **C-D**: Performance for the red-light pattern deconvolution under simultaneous projection after applying (**C**) n_neighbors = 20 or (**D**) n_neighbors = 10 to Local Outlier Factor. The pixels with value -1 (orange) were used to signify the inaccurate pixels when comparing with the true values. Note: For red-light pattern deconvolution, cluster grouping was executed by grouping the two clusters with lower means into a single ‘OFF’ cluster and the other cluster/s as ‘ON’ cluster.

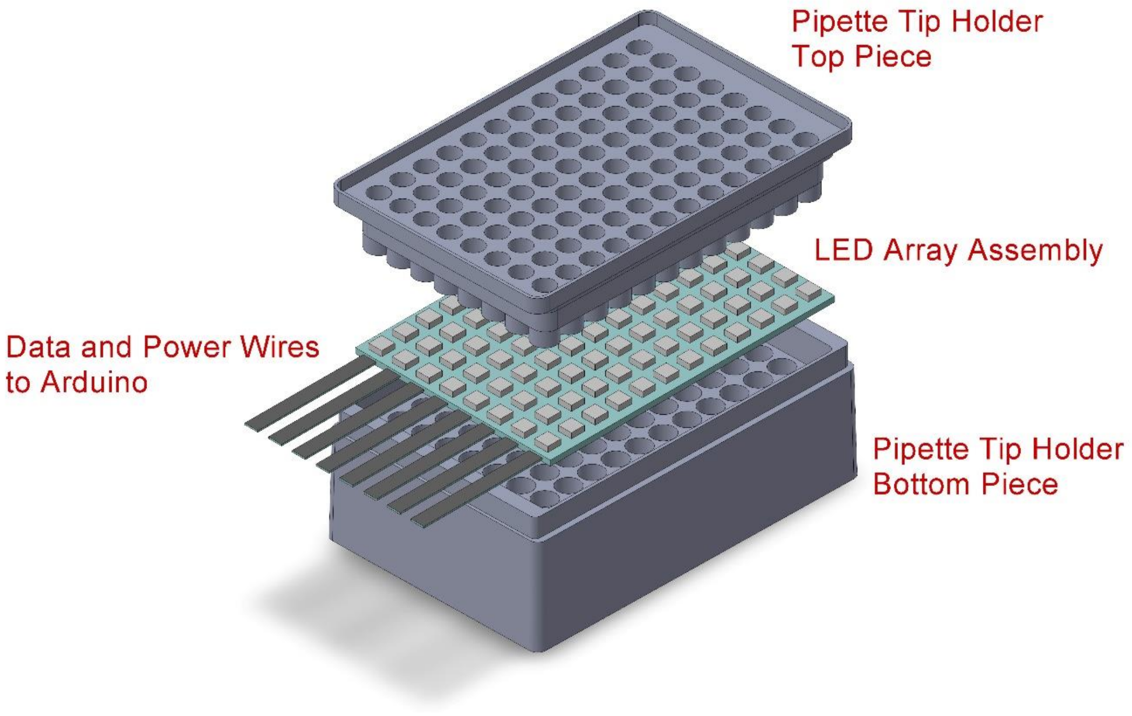

Supplementary S8: Visual illustration of a custom-built light illumination box (OptoBox). This is illustrated using AutoCAD.

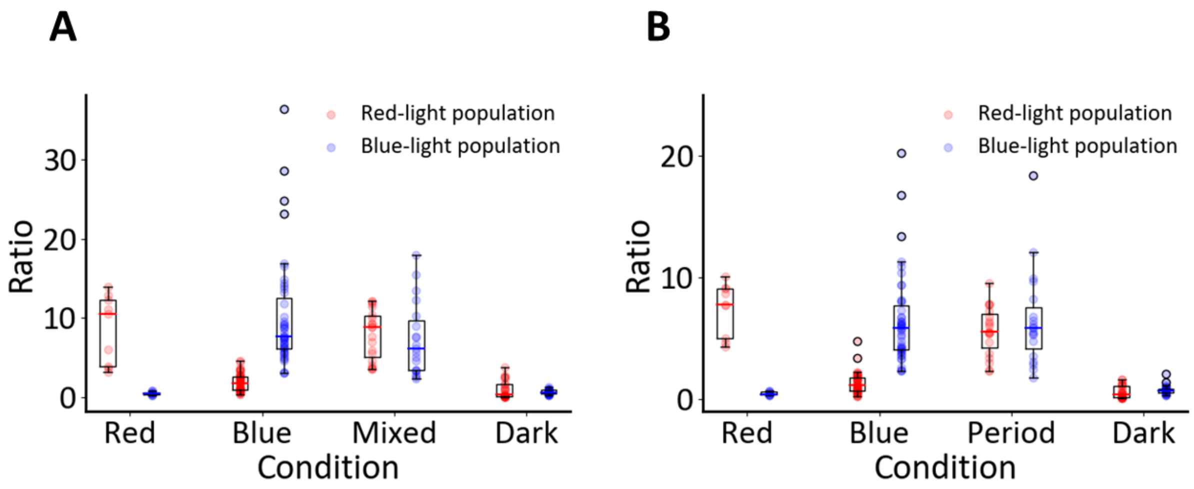

Supplementary S9: Orthogonality of the red and blue light systems. This figure is a re-representation of the data in Fig. 5. Box-and-whisker plots were plotted with a box extending from lower to upper quartile of data provided, with a line at the median. Whisker range is 1.5 time interquartile range. No statistics were derived for this figure. **A**: Performance of each system in differing light conditions when patterns of two light colors were projected simultaneously. It can be observed that the red light population was higher in 'Red' and 'Mixed'

light conditions, as compared to 'Blue' and 'Dark' conditions, whereby 'Mixed' indicates wells where both blue and red light were projected simultaneously. It can also be observed that the blue light population was higher in 'blue' and 'mixed' light conditions as compared to 'red' and 'dark', thus demonstrating orthogonality between both systems. **B**: Performance of each system in differing light conditions when patterns of two light colors were projected in a periodic and toggling fashion. Observations were similar to the results when light was projected simultaneously, whereby the 'Period' condition in this case indicates wells where both blue and red light were projected in a period fashion.

Supplementary Table 1. Encoded Images in Multiplexed Pool
Five images were encoded in a similar manner. Each image was tagged with a meta-barcode as per the table below:

NUS	PCR P7 N701-47u	P5-Index501-Read1-Fwd-45u
SynCTI	PCR P7 N705-47u	P5-Index501-Read1-Fwd-45u
Smiley	PCR P7 N701-47u	P5-Index503-Read1-Fwd-45u
BacCam	PCR P7 N705-47u	P5-Index503-Read1-Fwd-45u
Heloo Wo{|d!	PCR P7 N709-47u	P5-Index505-Read1- Fwd-45u

Supplementary Table 2. Accession numbers for the raw sequencing data

SRA Accession	BioProject Accession	BioSample Accession	Sample Name	URL
SRR24524113	PRJNA970212	SAMN34995462	Amplicon Seq BacCam Fig 1	https://www.ncbi.nlm.nih.gov/sra/SRX20308333
SRR24524110	PRJNA970212	SAMN35006154	Fig 2 Mix BacCam	https://www.ncbi.nlm.nih.gov/sra/SRX20308317
SRR24524109	PRJNA970212	SAMN35006155	Fig 2 Mix HW	https://www.ncbi.nlm.nih.gov/sra/SRX20308320
SRR24524108	PRJNA970212	SAMN35006164	Fig 2 RA Dilute 50x	https://www.ncbi.nlm.nih.gov/sra/SRX20308319
SRR24524107	PRJNA970212	SAMN35006165	Fig 2 RA Dilute 500x	https://www.ncbi.nlm.nih.gov/sra/SRX20308320
SRR24524106	PRJNA970212	SAMN35006166	Fig 2 RA Dilute 5000x	https://www.ncbi.nlm.nih.gov/sra/SRX20308321
SRR24524105	PRJNA970212	SAMN35006167	Fig 2 RA Dilute 50000x	https://www.ncbi.nlm.nih.gov/sra/SRX20308322
SRR24524104	PRJNA970212	SAMN35006156	Fig 2 Mix NUS	https://www.ncbi.nlm.nih.gov/sra/SRX20308323

SRR24524103	PRJNA970212	SAMN35006157	Fig 2 Mix Smiley	https://www.ncbi.nlm.nih.gov/sra/SRX20308324
SRR24524102	PRJNA970212	SAMN35006158	Fix 2 Mix SynCTI	https://www.ncbi.nlm.nih.gov/sra/SRX20308325
SRR24524101	PRJNA970212	SAMN35006159	Fig 2 RA BacCam	https://www.ncbi.nlm.nih.gov/sra/SRX20308326
SRR24524100	PRJNA970212	SAMN35006160	Fig 2 RA HW	https://www.ncbi.nlm.nih.gov/sra/SRX20308327
SRR24524099	PRJNA970212	SAMN35006161	Fig 2 RA NUS	https://www.ncbi.nlm.nih.gov/sra/SRX20308328
SRR24524098	PRJNA970212	SAMN35006162	Fig 2 RA Smiley	https://www.ncbi.nlm.nih.gov/sra/SRX20308329
SRR24524097	PRJNA970212	SAMN35006163	Fig 2 RA SynCTI	https://www.ncbi.nlm.nih.gov/sra/SRX20308330
SRR24524093	PRJNA970212	SAMN35017434	Supp S4 0	https://www.ncbi.nlm.nih.gov/sra/SRX20308309
SRR24524092	PRJNA970212	SAMN35017435	Supp S4 0.5	https://www.ncbi.nlm.nih.gov/sra/SRX20308310
SRR24524091	PRJNA970212	SAMN35017436	Supp S4 1	https://www.ncbi.nlm.nih.gov/sra/SRX20308311
SRR24524090	PRJNA970212	SAMN35017437	Supp S4 1.5	https://www.ncbi.nlm.nih.gov/sra/SRX20308312
SRR24524089	PRJNA970212	SAMN35017438	Supp S4 2	https://www.ncbi.nlm.nih.gov/sra/SRX20308313
SRR24524088	PRJNA970212	SAMN35009593	Fig 3 HW Frozen	https://www.ncbi.nlm.nih.gov/sra/SRX20308300
SRR24524087	PRJNA970212	SAMN35009594	Fig 3 HW Heat	https://www.ncbi.nlm.nih.gov/sra/SRX20308301
SRR24524086	PRJNA970212	SAMN35009595	Fig 3 HW RT	https://www.ncbi.nlm.nih.gov/sra/SRX20308302
SRR24524085	PRJNA970212	SAMN35009596	Fig 3 HW UV	https://www.ncbi.nlm.nih.gov/sra/SRX20308303
SRR24524084	PRJNA970212	SAMN35009597	Fig 3 Smiley 10X	https://www.ncbi.nlm.nih.gov/sra/SRX20308304
SRR24524083	PRJNA970212	SAMN35009598	Fig 3 Smiley 100X	https://www.ncbi.nlm.nih.gov/sra/SRX20308305
SRR24524082	PRJNA970212	SAMN35009599	Fig 3 Smiley 1000X	https://www.ncbi.nlm.nih.gov/sra/SRX20308306
SRR24524081	PRJNA970212	SAMN35009600	Fig 3 Smiley 1X	https://www.ncbi.nlm.nih.gov/sra/SRX20308307
SRR24524080	PRJNA970212	SAMN35009601	Fig 3 Smiley Dried	https://www.ncbi.nlm.nih.gov/sra/SRX20308308
SRR24524079	PRJNA970212	SAMN35017330	Fig 5 Mixed Light	https://www.ncbi.nlm.nih.gov/sra/SRX20308298
SRR24524078	PRJNA970212	SAMN35017331	Fig 5 Period Light	https://www.ncbi.nlm.nih.gov/sra/SRX20308299

R Script for deconvoluting reads

R script for deconvoluting reads is located in the Supplementary Software folder and named as ‘Script for Light Patterns Deconvolution’.

Python Script for clustering

Python code for clustering is located in the Supplementary Software folder and named as ‘Codes_Clustering_technique’.
