# Supplementary material for: A biological camera that captures and stores images directly into DNA
Source: Nat Commun. 2023 Jul 3;14:3921. doi: 10.1038/s41467-023-38876-w (PMC10318082; doi:10.1038/s41467-023-38876-w)
Supplement: Supplementary file 4 — Description of Additional Supplementary Files [file 41467_2023_38876_MOESM4_ESM.pdf]

Title: Supplementary Software

Description: The supplementary folder contains two folders/files.

The first is a compressed zip file named 'Codes_Clustering_technique'. Within the folder there is a folder named 'Output_figures' containing the figures generated by the python code from the input datasets, a python script for the clustering named 'Automated_Image_deconvolution_software.py', a datafile containing the output image from clustering named 'Datafile', as well as a markdown file named 'README.md' detailing the use of the script.

The second is a R script for deconvolution of reads from raw sequencing reads named 'Script for Light Patterns Deconvolution'.
